# Age at quitting smoking as a predictor of risk of cardiovascular disease incidence independent of smoking status, time since quitting and pack-years

**DOI:** 10.1186/1756-0500-4-39

**Published:** 2011-02-15

**Authors:** Haider R Mannan, Christopher E Stevenson, Anna Peeters, Helen L Walls, John J McNeil

**Affiliations:** 1 Department of Epidemiology & Preventive Medicine, Monash University, Melbourne, Victoria 3004, Australia

## Abstract

**Background:**

Risk prediction for CVD events has been shown to vary according to current smoking status, pack-years smoked over a lifetime, time since quitting and age at quitting. The latter two are closely and inversely related. It is not known whether the age at which one quits smoking is an additional important predictor of CVD events. The aim of this study was to determine whether the risk of CVD events varied according to age at quitting after taking into account current smoking status, lifetime pack-years smoked and time since quitting.

**Findings:**

We used the Cox proportional hazards model to evaluate the risk of developing a first CVD event for a cohort of participants in the Framingham Offspring Heart Study who attended the fourth examination between ages 30 and 74 years and were free of CVD. Those who quit before the median age of 37 years had a risk of CVD incidence similar to those who were never smokers. The incorporation of age at quitting in the smoking variable resulted in better prediction than the model which had a simple current smoker/non-smoker measure and the one that incorporated both time since quitting and pack-years. These models demonstrated good discrimination, calibration and global fit. The risk among those quitting more than 5 years prior to the baseline exam and those whose age at quitting was prior to 44 years was similar to the risk among never smokers. However, the risk among those quitting less than 5 years prior to the baseline exam and those who continued to smoke until 44 years of age (or beyond) was two and a half times higher than that of never smokers.

**Conclusions:**

Age at quitting improves the prediction of risk of CVD incidence even after other smoking measures are taken into account. The clinical benefit of adding age at quitting to the model with other smoking measures may be greater than the associated costs. Thus, age at quitting should be considered in addition to smoking status, time since quitting and pack-years when counselling individuals about their cardiovascular risk.

## Introduction

CVD risk associated with smoking varies not only with smoking status but also with intensity and duration of smoking or smoking pack-years, time since quitting and age at quitting. Many studies have examined the lag in health benefit of smoking cessation measured by time since quitting and occurrence of a CVD event [1-8]. However age at quitting also affects the health benefits of smoking cessation [9,10]. The risks of mortality and smoking-related disease increase with age at quitting. However, the role of age at quitting as a predictor of CVD risk in the presence of time since quitting and pack-years is unclear. As CVD is more common among elderly people it is likely that age at quitting and time since quitting, inversely correlated, influence CVD risk in opposite directions.

This study explores whether and how age at quitting influences risk of CVD incidence, using data from the Framingham Offspring Heart Study. It uses a smoking status variable with and without incorporating other smoking variables such as time since quitting among past smokers and pack-years among current smokers, while controlling for common risk factors.

## Methods & Results

### Study Design and Sample

The Framingham Offspring Heart Study details for design, selection criteria, examination procedures and criteria for CVD events have been described elsewhere [11-15]. Participants were eligible for the present study if at examination 4 (1988 to 1992) they were CVD-free and aged 30-74 with nonmissing data on covariates. The final study sample consisted of 3751 participants (mean age 51.61; 1937 women).

### Measurement of CVD Risk Factors

The risk factors included were smoking status with various definitions (expanded below), systolic and diastolic blood pressure (SBP & DBP), total cholesterol/high-density-lipoprotein (HDL) ratio or both (depending on which provided a better prediction of outcome), age, sex, diabetes status and body-mass index (BMI). Smoking status was initially defined as a dichotomous current smoker/non-smoker variable. The other definitions of smoking status included four, six and eight categories. The four category smoking status variable was defined as: never smokers, former smokers with age at quitting below 37 years, former smokers with age at quitting of 37 years or older, and current smokers. The six category smoking status variable was defined as: never smokers, former smokers with time since quitting 5 years or less and over 5 years and current smokers with under 20, 20-39, and 40 or more pack-years where current pack-years were calculated by dividing the number of cigarettes being smoked per day by 20 to obtain an estimate of "packs" and multiplying this by the number of years a person was a smoker. The eight category smoking status variable was defined as: never smokers, past smokers into 4 groups with two levels of age at quitting (≤44, >44 years) at each of two levels of time since quitting (≤5, and >5 years) and current smokers with under 20, 20-39, and 40 or more pack-years.

Blood pressure was the average of two physician-obtained measures. Cholesterol and various smoking measures were based on standardized enzymatic methods and self-report, respectively. Diabetes was defined as a fasting glucose ≥126 mg/dL. Age at quitting and time since quitting were calculated at examination 4 by combining smoking status information at each examination with history of smoking status from examination 1 [8].

### Development & assessment of predictive models

The Cox proportional-hazards model [16] was used to relate risk factors to the risk of CVD incidence during follow-up from examinations 4 to 7. The assumption of proportionality of hazards was satisfied; tested by taking interaction between a covariate and log (survival time) [17] and plotting Schoenfeld residuals against survival time.

To improve the interpretability of the predictive models we categorized time since quitting, age at quitting and pack-years. It was observed that the lag time for a beneficial effect of smoking cessation on risk of CVD incidence was five years after which the risk stabilized [8]. In the literature there is no maximum age for quitting without increasing the risk of a CVD event compared to a never smoker and there was no apparent cutpoint to dichotomise this variable as the predicted time to the onset of a CVD event declined almost linearly with age at quitting (results not shown). Thus the median, shown by simulation to result in minimum loss in efficiency [18], was used to dichotomise age at quitting.

Four models were fitted for the outcome. Each included a composite measure of smoking status and all other risk factors found to be significantly related to the outcome. Model 1 included smoking status as a simple current smoker/non-smoker variable with current non-smoker as the reference category. To incorporate the effect of age at quitting into smoking status, quitters were separated from current non-smokers and categorized by age at quitting in Model 2, which incorporated smoking status with categories <37 and ≥37 years for age at quitting, never smokers and current smokers. To examine whether incorporating age at quitting to smoking status improved risk prediction, Model 2 was compared to Model 1. To examine whether incorporating age at quitting improves risk prediction to a model which already includes time since quitting and pack-years in smoking status, Models 3 and 4 were fitted and compared. Model 3 incorporated smoking status that included categories for never smokers, ≤5 and >5 years for time since quitting, and <20, 20-39 and 40+ for pack-years. Other categorizations for pack-years and time since quitting were found to be less effective in terms of predictive ability. Model 4 added age at quitting to Model 3 with smoking status having six categories - never smoker, current smoker, and past smokers quitting ≤5 years and whose age at quitting was ≤ 44 or >44 years, and those quitting >5 years and whose age at quitting was ≤ 44 or >44 years. Compared with Model 2 age at quitting was categorized differently because for the initial categorization of age at quitting at < 37 and ≥37 years there were inadequate numbers of cases in one of the joint categories of this variable with time since quitting resulting in inefficient estimation of its regression coefficient. The other cutpoints prior to reaching age 44 produced the same result until the cutpoint reached age 44 which did not yield inadequate number of cases in any of the joint categories. In Models 2 through 4 the reference category for smoking status was never smoker.

For assessing the discriminative ability of a model and improvement between two nested models we used Harrell's c statistic [19,20] and a test for difference in two correlated c statistics [21]. Large 'independent' association of the new covariate with the outcome is required to result in a meaningfully larger c statistic [22-24] for models possessing reasonably good discrimination, and the c statistic does not assist a physician in treatment decisions about an individual [25,26] while reclassification statistics NRI [27] and IDI [27] do [25,26]. Thus, we used the latter to supplement c-statistic analyses [27,28]. For calculating NRI we assessed risk reclassification [27] by sorting the predicted risk for each model into four clinically meaningful categories (<6%, 6% to < 10%, 10% to < 20%, and ≥ 20%). The benefit and cost of using a new model compared to a baseline model can be measured by the proportions of subjects with and without subsequent events, respectively, who are classified as high risk (eg. ≥ 20%) according to the new model [26]. There was negligible overoptimism in c and NRI estimates obtained by bootstrapping as these were less than 0.007 and 0.005 respectively.

For assessing calibration of the fitted models and improvement in global fit between two nested models we computed the Hosmer-Lemeshow statistic and its modification [28] and likelihood ratio test respectively. Neither Models 3 nor 4 included current age as a covariate because it had exact collinearity with time since quitting and age at quitting.

## Results

### Sample characteristics

The sample risk factor characteristics at baseline examination 4 are shown in Table 1. The sample consists of 26.7% never smokers, 48.4% quitters (15.1% of whom quit within 5 years of the baseline measurement and 33.1% of whom quit before age 37 years), and 14.5% current smokers (of whom 16.6% have exposure ≥40 pack-years).

**Table 1:** Summary Statistics for Risk Factors (at exam 4) Used in Risk Models for Total Population Characteristics

	Summary statistic
**Sex, N (%)**	
- **Females**	1937 (51.6)
- **Males**	1814 (48.4)
**Age (years), mean (SD)**	51.61 (9.6)
**Total-C (mg/dL), mean (SD)**	206.30 (39.2)
**HDL-C (mg/dL), mean (SD)**	49.52 (14.8)
**Systolic blood pressure (mmHg), mean (SD)**	127.11 (18.9)
**Diastolic blood pressure (mmHg), mean (SD)**	79.25 (10.0)
**Triglycerides (meq/liter), mean (SD)**	125.92 (101.3)
**Alcohol (ounce), mean (SD)**	2.89 (4.4)
**Total cholesterol (mg/dl), mean (SD)**	206.31(39.2)
**HDL cholesterol (mg/dl), mean (SD)**	49.52 (14.8)
**Total/HDL cholesterol ratio, mean (SD)**	4.54 (1.7)
**Body mass index (kg/m**^**2**^**), mean (SD)**	26.86 (4.8)
**Never smoking, N (%)**	1002 (26.7)
**Past smoking, N (%)**	1817 (48.4)
**Time since quitting ≤ 5 years, N (%)**	565 (15.1)
**Time since quitting >5 years, N (%)**	1252 (33.4)
**Age at quitting <37 years, N (%)**	1240 (33.1)
**Age at quitting > = 37 years, N (%)**	577 (15.4)
**Current smoking, N (%)**	932 (24.9)
**Pack years <20, N (%)**	105 (2.8)
**Pack years 20-39, N (%)**	206 (5.5)
**Pack years 40+, N (%)**	621 (16.6)
**Diabetes, N (%)**	163 (4.4)

### Model comparisons

Table 2 shows that Model 2 improved predictive ability significantly compared to Model 1. Model 3 performed well in terms of model discrimination and overall fit but less well in terms of calibration. Model 4 performed well on all model performance indicators; significantly improving predictive ability compared to Model 3 (Table 3). Thus, age at quitting was an independent predictor of risk of CVD incidence regardless of including time since quitting and pack-years in the model.

**Table 2:** Improvement in CVD risk prediction due to including age at quitting among past smokers in Model 1

Likelihood ratio	Value	Degrees of freedom		p-value
**Vs model 1**	11.4732	2		0.0032

**Difference between two correlated C**	**Estimate (SE)**	**95% CI**	**Chi-square**	**p-value**

**Vs model 1**	0.0047(0.0022)	0.0004, 0.0090	4.5340	0.0332

	**Estimate**	**95% CI**	**Z**	**p-value**

**NRI**				
**Vs model 1**	0.0512	0.0117, 0.0906	2.5343	0.0113
**IDI**				
**Vs model 1**	0.0014	-0.0010,0.0037	1.1012	0.2707

Note: Model 1 included current smoking status, systolic and diastolic blood pressure, total cholesterol/HDL ratio, triglycerides, age, sex and diabetes status.

**Table 3:** Improvement in CVD risk prediction due to including age at quitting among past smokers in Model 3

Likelihood ratio	Value	Degrees of freedom		p-value
**Vs model 3**	25.8845	2		<0.0001

**Difference between two correlated C**	**Estimate (SE)**	**95% CI**	**Chi-square**	**p-value**

**Vs model 3**	0.0079(0.0036)	0.0008,0.0150	4.7266	0.0297

	**Estimate**	**95% CI**	**Z**	**p-value**

**NRI**				

**Vs model 3**	0.0294	-0.0111,0.0701	1.4192	0.1558
**IDI**				
**Vs model 3**	0.0029	0.0001, 0.0057	2.0362	0.0417

Note: Model 3 incorporated smoking status that included categories for never smokers (reference group), ≤5 and >5 years for time since quitting, and <20, 20-39 and 40+ for pack-years, systolic and diastolic blood pressure, total cholesterol/HDL ratio, triglycerides, age, sex and diabetes status.

Compared to never smokers, the risk of CVD incidence based on Model 2 was 7.3% higher (RR = 1.073, 95% CI 0.804 ~ 1.433) for those who quit before age 37 years and 58.1% higher (RR = 1.581, 95% CI 1.193 ~ 2.094) for those who quit at least at age 37 years (Table 4). For the former category the relative risk was not significantly different from the never smokers while for the latter category it was. Based on the final model (Model 4), the risk among those quitting more than 5 years prior to the baseline exam and whose age at quitting was 44 years or less was close to never smokers. Risk among those quitting within 5 years prior to the baseline exam and whose age at quitting was over 44 years was about three times higher than that of never smokers (Table 5).

**Table 4:** Risk equation with a simple current/non-smoker smoking status variable (Model 1)

Variable	Parameter Estimate	Standard Error	Chi-Square	P value	Hazard Ratio	95% CI	
Sex	0.6391	0.1119	32.6222	<.0001	1.895	1.522	2.360
Age	0.0768	0.0069	123.1031	<.0001	1.080	1.065	1.095
Sbp	0.0142	0.0036	14.9743	0.0001	1.014	1.007	1.022
Dbp	-0.0176	0.0069	6.3866	0.0115	0.983	0.969	0.996
Total/HDL ratio	0.2127	0.0347	37.4452	<.0001	1.237	1.156	1.324
Diabetes	0.8101	0.1518	28.4764	<.0001	2.248	1.670	3.028
Trglycerides	-0.0015	0.0005	7.2971	0.0069	0.998	0.997	1.000
Current smoker	0.5594	0.1251	19.9779	<.0001	1.750	1.369	2.236

Test	Total	Event		Censord			% Censord
	3751	383		3368			89.79
							
	Chi-Square	DF				P value	
Likelihood Ratio	437.1565	8				<. 0001	
Hosmer Lemeshow	18.6207	9				0.0286	
Modified HL	17.4569	9				0.0420	
							
	Estimate	SE				95% CI	
C statistic	0.8038	0.0109				0.7825,0.8251	

Note: The reference categories for sex, diabetes and the smoking variable are female, no diabetes and non-current smoker respectively.

**Table 5:** Risk equation with age at quitting incorporated into smoking status variable (Model 2)

Variable	Parameter Estimate	Standard Error	Chi-Square	P value	Hazard Ratio	95% CI	
Sex	0.5967	0.1133	27.7286	<.0001	1.816	1.454	2.268
Age	0.0730	0.0070	107.9667	<.0001	1.076	1.061	1.091
Sbp	0.0140	0.0036	14.7801	0.0001	1.014	1.007	1.021
Dbp	-0.0172	0.0069	6.1574	0.0131	0.983	0.970	0.996
Total/HDL ratio	0.2148	0.0354	36.7663	<.0001	1.240	1.157	1.329
Diabetes	0.7941	0.1520	27.2897	<.0001	2.213	1.642	2.981
Trglycerides	-0.0016	0.0005	8.2076	0.0042	0.998	0.997	0.999
Current smoker	0.7248	0.1505	23.1684	<.0001	2.064	1.537	2.773
Age at quitting							
<37	0.0708	0.1474	0.2307	0.6310	1.073	0.804	1.433
> = 37	0.4578	0.1435	10.1778	0.0014	1.581	1.193	2.094

Test	Total	Event		Censored			% Censored
	3751	383		3368			89.79
							
	Chi-Square	DF				P value	
Likelihood Ratio	448.6297	10				<.0001	
Hosmer Lemeshow	11.3628	9				0.2516	
Modified HL	10.5026	9				0.3113	
							
	Estimate	SE				95% CI	
C statistic	0.8085	0.0108				0.7873, 0.8296	

Note: The reference categories for sex, diabetes and the smoking variable are female, no diabetes and never smoker respectively.

### Reclassification of subjects

This section describes how many subjects were reclassified overall and with respect to 'high risk' category of ≥ 20% when we compared the preferred full model against the reference model. Comparing Model 2 against Model 1, for participants who experienced a CVD event, the net gain in reclassification proportion was significantly different from zero (p = 0.0113) (Table 6) and significant for participants who did not experience an event (p = 0.0025) and for all participants (p = 0.0112). For those who experienced a CVD event, using Model 4 rather than Model 3 did not improve net gain in reclassification proportion significantly (p = 0.2935) (Table 7). The result was similar for participants who did not experience an event (p = 0.1545) and for all participants (p = 0.1558).

**Table 6:** Risk equation incorporating time since quitting and pack-years into smoking status (Model 3)

Variable	Parameter Estimate	Standard Error	Chi-Square	P value	Hazard Ratio	95% CI	
Sex	0.66172	0.11252	34.5854	<.0001	1.938	1.555	2.416
Sbp	0.03508	0.00306	131.2240	<.0001	1.036	1.030	1.042
Dbp	-0.04076	0.00621	43.1167	<.0001	0.960	0.948	0.972
Total/HDL ratio	0.22727	0.03592	40.0381	<.0001	1.255	1.170	1.347
Diabetes	0.88176	0.15423	32.6873	<.0001	2.415	1.785	3.268
Trglycerides	-0.00170	0.00059	8.2356	0.0041	0.998	0.997	0.999
Time since quitting							
< = 5 years	0.75854	0.18123	17.5189	<.0001	2.135	1.497	3.046
>5 years	0.13762	0.13059	1.1106	0.2920	1.148	0.888	1.482
Pack years							
<20	0.15632	0.34319	0.2075	0.6487	1.169	0.597	2.291
20-39	0.50078	0.21593	5.3785	0.0204	1.650	1.081	2.519
40+	0.81008	0.16219	24.9470	<.0001	2.248	1.636	3.089

Test	Total	Event		Censored			% Censored
	3751	383		3368			89.79
							
	Chi-Square	DF				P value	
Likelihood Ratio	324.1494	11				<0.0001	
Hosmer Lemeshow	11.4700	9				0.2448	
Modified HL	11.0293	9				0.2737	
							
	Estimate	SE				95% CI	
C statistic	0.7601	0.0130				0.7346, 0.7856	

Note: The reference categories for sex, diabetes and the smoking variable are female, no diabetes and never smoker respectively.

**Table 7:** Risk equation for CVD incidence incorporating age at quitting, time since quitting & pack-years into smoking status (Model 4)

Variable	Parameter Estimate	Standard Error	Chi-Square	P value	Hazard Ratio	95% CI	
Sex	0.66579	0.11303	34.6954	<.0001	1.946	1.559	2.429
Sbp	0.03269	0.00315	107.7472	<.0001	1.033	1.027	1.040
Dbp	-0.03884	0.00632	37.8124	<.0001	0.962	0.950	0.974
Total/HDL ratio	0.22400	0.03635	37.9841	<.0001	1.251	1.165	1.343
Diabetes	0.85100	0.15482	30.2145	<.0001	2.342	1.729	3.172
Trglycerides	-0.00185	0.00061	9.1876	0.0024	0.998	0.997	0.999
Pack-years							
< = 19	0.15082	0.34314	0.1932	0.6603	1.163	0.593	2.278
20-39	0.48640	0.21593	5.0743	0.0243	1.626	1.065	2.483
40+	0.81617	0.16208	25.3579	<.0001	2.262	1.646	3.108
Time since quitting							
< = 5 years & Age at quitting							
≤ 44	-0.9483	0.71256	1.7711	0.1832	0.387	0.096	1.566
>44	1.0505	0.18672	31.6533	<.0001	2.859	1.983	4.123
Time since quitting							
>5 years &							
Age at quitting							
≤ 44	-0.1047	0.1595	0.4315	0.5113	0.901	0.659	1.231
>44	0.5923	0.1789	10.9580	0.0009	1.808	1.273	2.568

Test	Total	Event		Censored			% Censored
	3751	383		3368			89.79
							
	Chi-Square	DF				P value	
Likelihood Ratio	347.7915	13				<.0001	
Hosmer Lemeshow	6.5337	9				0.6855	
Modified HL	6.1388	9				0.7259	
							
	Estimate	SE				95% CI	
C statistic	0.7680	0.0130				0.7426, 0.7934	

Note: The reference categories for sex, diabetes and the smoking variable are female, no diabetes and never smoker respectively.

Table 8 shows that based on Model 2 instead of Model 1, 16.5% of those developing a CVD event would have moved up to the 'high risk' category of ≥20% while of those not having a CVD event 9.4% would have moved to this risk category, the difference of which is highly significant (p < 0.0001). Similarly, Table 9 shows that if we had used Model 4 rather than Model 3, 14.6% of those who develop CVD would be appropriately assessed for their cardiovascular risk while only 7.6% of those who do not develop CVD would be falsely assessed for their cardiovascular risk, the difference of which is highly significant (p < 0.0001).

**Table 8:** Reclassification table for risk of CVD incidence between the model with age at quitting incorporated into smoking status (Model 2) and the model with a current/non-smoker smoking measure (Model 1) as the reference model

Model 1	Model 2				
**Frequency (Row per cent)**	***<*6%**	**6-<10%**	**10-<20%**	**> = 20%**	**Total**

Participants who experience a CVD Event					

<6%	158	16	0	0	174

6-<10%	8	53	12	0	73

10-<20%	0	5	60	6	71

> = 20%	0	0	8	57	65

Total	166	74	80	63	383

Net gain in reclassification proportion (p-value)	0.0339(0.0796)				

Participants who do not experience a CVD Event					

<6%	1750	53	0	0	1803

6-<10%	75	469	67	0	611

10-<20%	0	79	499	36	614

> = 20%	0	0	60	280	340

Total	1825	601	626	316	3368

Net gain in reclassification proportion (p-value)	0.0172(0.0025)				

NRI (p-value)	0.0511(0.0112)				

Overall net gain in reclassification proportion with respect to risk category > = 20%(p-value)	0.0019(0.8518)				

Overall gross gain in reclassification proportion with respect to risk category > = 20%(p-value)	0.0263(<0.0001)				

**Table 9:** Reclassification table for risk of CVD incidence between the model with age at quitting, time since quitting and pack-years incorporated into smoking status (Model 4) and a reduced model without age at quitting (Model 3) as the reference model

Model 3	Model 4				
**Frequency (Row per cent)**	***<*6%**	**6- < 10%**	**10- < 20%**	**> = 20%**	**Total**

Participants who experience a CVD Event					

<6%	164	13	0	0	177

6- < 10%	10	56	12	0	78

10- < 20%	1	5	57	8	71

> = 20%	1	0	8	48	57

Total	176	74	77	56	383

Net gain in reclassification proportion (p-value)	0.0208(0.2935)				

Participants who do not experience a CVD Event					

<6%	1402	85	0	0	1487

6- < 10%	83	742	64	0	889

10- < 20%	25	84	596	44	749

> = 20%	10	2	18	213	243

Total	1520	913	678	257	3368

Net gain in reclassification proportion (p-value)	0.0086(0.1545)				

NRI (p-value)	0.0294(0.1558)				

Overall net gain in reclassification proportion with respect to risk category > = 20%(p-value)	0.0015(0.8888)				

Overall gross gain in reclassification proportion with respect to risk category > = 20%(p-value)	0.0339(<0.0001)				

### Sensitivity of the results

We have adjusted for all major confounders of smoking to address confounding bias in the risk models. To address the possibility of distortion due to medical treatments affecting the risk of a CVD event we found that the regression coefficients of the models were fairly insensitive to the inclusion of cardioactive medications. To address the possibility of reverse causation, we excluded from the baseline cohort those with a cancer history and other non-CVD conditions. This did not substantially influence the results. Sub-analyses conducted by excluding from baseline cohort those smokers who quit after examination 4 and those quitters who took up smoking after examination 4, and later those current smokers from baseline cohort whose pack-years changed substantially in subsequent examinations did not influence our results.

### Merits and demerits of this study

The study's key strength is that it not only evaluates improvement in predicting CVD risk when models incorporate age at quitting but also quantifies the proportions of people receiving clinical benefits and costs. However, a cost-benefit analysis of including this variable was not possible as the same number of CVD events was prevented by the full and reduced models. Also, as the Framingham cohort has an ethnically white predominance the generalizability of our models to other ethnic groups is unknown.

## Conclusion

The incorporation of age at quitting in smoking status resulted in better prediction compared to the model which had a current smoker/non-smoker measure and to the model which incorporated both time since quitting and pack-years in smoking status. Thus, age at quitting was an independent predictor of CVD incidence even after accounting for time since quitting and pack-years.

We also showed that if we had incorporated age at quitting in smoking status instead of a current/non-smoker measure, a significantly higher proportion of those developing a CVD event would have moved up to the 'high risk' category compared to those not having a CVD event who moved up to this category. The result was similar if the model added age at quitting in smoking status which already incorporated time since quitting and pack-years. The former would be appropriately treated while the latter would be falsely treated if we included age at quitting in smoking status. Those appropriately treated can benefit from additional screening for CVD risk and would require more aggressive intervention for smoking cessation [29] and would thus aid in preventing more deaths. However, this benefit would be at the cost of falsely identifying people who do not develop CVD as high risk who may unnecessarily receive additional screening and may cause undue stress and burden to the smoking cessation programs. From a CVD prevention perspective the benefits associated with smoking cessation clearly outweigh the costs for CVD screening and smoking cessation programs. Age at quitting should be taken into account, as well as other smoking measures, when counselling individuals about their cardiovascular risk.

## Competing interests

The authors declare that they have no competing interests.

## Authors' contributions

HRM was involved in all stages of this research as the principal author. CES, AP and HLW read the draft of the paper and provided useful suggestions. JJM is the Principal Investigator of the grant which enabled this research to be carried out. All authors read and approved the final manuscript.
